# Covalent Immobilization of Different Enzymes on Hydroxyapatite, an Alternative Green Support

**DOI:** 10.1021/acsomega.5c06430

**Published:** 2025-09-01

**Authors:** Leonardo Gelati, Antonella Gervasini, Giovanna Speranza, Francesca Paradisi

**Affiliations:** † Department of Chemistry, 9304 University of Milan, via C. Golgi 19, 20139 Milan, Italy; ‡ Department of Architecture and Industrial Design, University of Campania Luigi Vanvitelli, via San Lorenzo—Abazia di San Lorenzo, 81031 Aversa, Italy; § Department of Chemistry, Biochemistry and Pharmacology, 27210 University of Bern, Freiestrasse 3, 3012 Bern, Switzerland

## Abstract

Greener and cheaper alternatives to petrol-based supports have been studied in recent years to implement biocatalysis in industrial processes exploiting enzyme immobilization. Among these, hydroxyapatite (HAP) represents a suitable candidate thanks to its structural stability, nontoxicity, large surface area, and ease of surface modification. As it can be sourced from waste, it also fulfills the circular economy principles. This work explored the use of HAP for covalent immobilization using three model enzymes: a vanadium-dependent chloroperoxidase from *Curvularia inaequalis* (*Ci*VCPO), an l-tyrosine decarboxylase from *Lactobacillus brevis* (*Lb*TDC), and an *R*-selective transaminase from *Thermomyces stellatus* (*Ts*RTA). Different strategies were tested, and derivatization with (3-aminopropyl)­triethoxysilane (APTES) followed by glutaraldehyde activation was found to be the most widely applicable. *Lb*TDC and *Ts*RTA immobilized through this strategy were tested in multiple reaction cycles to assess their stability and reusability, with promising results.

## Graphical abstract

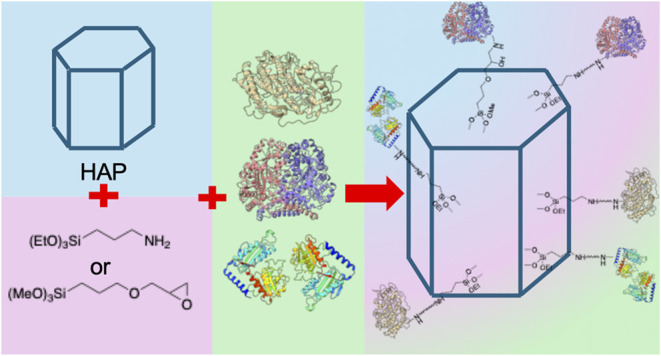

## Introduction

Enzyme immobilization has been recognized in recent years as a key technology to implement biocatalysis at the industrial level. This process, consisting of binding the protein on a support which is insoluble in the reaction solvent, despite usually lowering the enzymatic activity, provides an increase in protein stability under adverse conditions, such as extreme pHs, temperatures, and the presence of organic solvents, and allows the possibility of recycling the catalyst multiple times, leading to an overall decrease in process costs.^1^ Among all of the possible immobilization techniques, the formation of covalent bonds between the support and the enzyme grants higher stabilities and shelf lives of the catalyst.^2^

Moreover, the choice of the carrier must be carefully investigated, since it can greatly influence the properties of the final catalyst. In general, a good candidate material for enzyme immobilization should present a high surface area, be insoluble under the intended reaction conditions, chemically, structurally, and thermally stable, and, in order to facilitate its application at an industrial level, it should be cheap and environmentally friendly.^3^ Calcium hydroxyapatite (HAP, Ca_10_(PO_4_)_6_(OH)_2_) is a suitable candidate to be used as a support in enzyme immobilization thanks to its insolubility in water (*K*_PS_ ≈ 2.34 × 10^–59^), high mechanical strength, and surface area (about 100 m^2^ g^–1^). As the main inorganic component in mammalian bone tissue, it is also biocompatible. For these reasons, HAP has been studied since the ‘90s as a carrier for the immobilization of various enzymes, but most of the work reported in literature makes use of direct adsorption of the protein on the support, exploiting the ability of glutamic and aspartic acid residues to chelate calcium ions or other metals.^4,5^ The covalent bonding of enzymes on hydroxyapatite has been reported as well, but to a minor extent and with a limited number of strategies.^6^ The most applied strategy reported in literature for covalent immobilization on HAP, which has been also applied to silica supports,^7^ consists in a first derivatization of the inorganic support with (3-aminopropyl)­triethoxysilane (APTES) providing a handle with a primary amino group that is then reacted with glutaraldehyde that acts as a linker between the carrier and the lysine residues of the enzyme.^8^

In this work, we investigated three different strategies for covalent immobilization on hydroxyapatite and tested them with three different model enzymes for which literature precedence on immobilization was available for comparative purposes.^9–11^

The first enzyme, a vanadium-dependent chloroperoxidase from *Curvularia inaequalis* (*Ci*VCPO, E.C. 1.11.1.10), is a monomeric protein of cylindrical shape with a length of about 80 Å and a diameter of 55 Å.^12^ It catalyzes the production of hydrogen hypochlorite or hypobromite that can be used for different reactions such as halogenation of alkenes and arenes,^13^ haloether synthesis,^14^ halohydrin formation,^15^ aza-Achmatowicz reaction,^16^ halocyclization reactions,^17^ and many others.^18^

The second one is a tyrosine decarboxylase from *Lactobacillus brevis* (*Lb*TDC, E.C. 4.1.1.25). *Lb*TDC is a pyridoxal-5′-phosphate (PLP)-dependent enzyme with an asymmetric homodimeric structure. Its main activity is expressed in the decarboxylation reaction of l-tyrosine to produce tyramine, but it can also convert l-DOPA into dopamine.^19^

Finally, the *R*-selective transaminase from *Thermomyces stellatus* (*Ts*RTA) was selected. This enzyme is present in solution in an equilibrium between its dimeric and tetrameric forms and shows a good substrate scope for both the amine donor and the acceptor with high enantioselectivities of the product.^20^

## Materials and Methods

### General

All reagents were purchased from Sigma-Aldrich and used without further purification.

The plasmids of the three enzymes are available in our collection.

Computational analyses of the protein surface area composition were carried out using the CapiPy online tool,^21^ which exploits the homonymous software.^22^

^1^H NMR spectra were recorded on a Bruker spectrometer operating at 300 MHz. ^1^H NMR data recorded in DCl 7% wt. in D_2_O are listed as residual internal HDO (δ 4.79), and data recorded in DCl 7% wt. in (CD_3_)_2_SO are listed as residual internal (CD_2_H)­SO­(CD_3_) (δ 2.50).

Ultraviolet (UV) measurements were performed by using a Cary 60 UV–vis spectrophotometer (Agilent, Santa Clara, CA, USA).

High-performance liquid chromatography (HPLC) analyses were carried out through a Liquid Chromatography UHPLC system equipped with a binary pump, a diode array detector (Dionex UltiMate 3000, Thermo Scientific, Waltham, MA, USA), and an X-Bridge BEH C18 column (3.5 μm, 2.1 mm × 100 mm) (Waters, Elstree, U.K.). Eluent A was 0.1% TFA in water, and eluent B was methanol. The flow rate was set at 1 mL/min, the column was thermostated at 45 °C, and the following gradient was used: 0–1 min 1% B, 1–7 linear gradient to 40% B, 7–9 linear gradient to 45% B, and 9–10 linear gradient to 1% B. Detection was set at 280 nm.

All samples were dissolved in HCl (0.2% wt.) and filtered with a 0.45 μm nylon filter before injection.

### Hydroxyapatite Synthesis

The synthesis of hydroxyapatite (HAP) was performed following a literature-reported method.^23^

Briefly, (NH_4_)_2_HPO_4_ solution (0.1 M in degassed Milli-Q water, 250 mL) was placed in a five-necked 1 L round-bottom flask under nitrogen atmosphere and heated to 80 °C, and NH_4_OH (30%, 50 mL) was added. Then a Ca­(NO_3_)_2_·4H_2_O solution (0.167 M in degassed Milli-Q water, 250 mL) was added under stirring through a peristaltic pump (flow rate 1.67 mL min^–1^). A white solid started to precipitate.

Every 30 min, NH_4_OH (30%, 10 mL) was added to maintain the pH around 10. The reaction was stirred at 80 °C for 2 h and 30 min and then left aging under stirring for 5 min.

Hot filtration under reduced pressure was followed by washes with hot Milli-Q water until the filtrate reached a neutral pH (ca. 4 × 500 mL). The obtained white solid was dried at 70 °C in vacuo for 16 h and then at 120 °C for 8 h. The dried solid was ground to obtain homogeneous particle size, leading to a white powder.

To obtain larger particle sizes, the powder was first pressed using a hydraulic press; the resulting wafer was then ground and sieved using woven wire mesh stainless steel sieves by Endecotts, to obtain a homogeneous particles in the size range of 180–250 μm.

### Hydroxyapatite Functionalization with (3-Aminopropyl)­triethoxysilane (APTES)

Hydroxyapatite (500 mg, particle size: 180–250 μm) was put in a 15 mL Falcon tube and suspended in neat APTES (2 mL). The suspension was mixed at room temperature for 16 h by using an orbital shaker.

Then, the mixture was diluted with dichloromethane (2 mL), and the supernatant was removed and discarded. The solid was washed with dichloromethane (3 × 2 mL) and left to dry in the air for 4 h.

^1^H NMR (300 MHz, DCl 7% wt. in D_2_O): δ 0.26–0.027 (m, 2H), 0.52 (t, *J* = 7.10, 0.9H), 1.26–1.04 (m, 2H), 2.50–2.33 (m, 2H), 3.00 (q, *J* = 7.10 Hz, 0.6H) (see Figure S1).

### Hydroxyapatite Functionalization with (3-Glycidoxypropyl)­trimethoxysilane (GLYMO)

Hydroxyapatite (500 mg, particle size: 180–250 μm) was put in a 15 mL Falcon tube and suspended in neat GLYMO (2 mL). The suspension was mixed at room temperature for 16 h using an orbital shaker.

Then, the mixture was diluted with dichloromethane (2 mL), and the supernatant was removed and discarded. The solid was washed with dichloromethane (3 × 2 mL) and left to dry in air for 4 h.

^1^H NMR (300 MHz, DCl 7% wt. in *d*_6_-DMSO): δ 0.29 (br, 2H), 1.32 (br, 2H), 2.96 (s, 0.8H), 3.14 (br, 4H), 3.33 (br, 1H), 3.38 (br, 1H), 3.61 (br, 1H) (see Figure S2).

### Enzyme Expression and Purification

All three enzymes were expressed following previously reported protocols with some modifications.^20,24,25^ For additional information about sequence and structure analysis, see Figures S3–S8. Briefly, single colonies of recombinant *Escherichia coli* containing the plasmid coding for the enzyme (Arctic Express strain was used to express *Ci*VCPO cloned into a pBAD vector, BL21 (DE3) for *Lb*TDC harbored in a pRSETb vector, and BL21 STAR (DE3) for *Ts*RTA harbored in pET22b­(+)) were inoculated into the proper medium (LB for *Ci*VCPO, LB + d-glucose 1% wt. for *Lb*TDC, and TB + lactose 5 g/L for *Ts*RTA) supplemented with ampicillin (0.1 mg/mL) and incubated at 37 °C and 150 rpm for a certain amount of time (4 h in the case of *Ts*RTA, until reaching an OD_600_ between 0.6 and 0.8 cells/mL for the other enzymes). Then, a suitable inducer was added (l-arabinose 0.02% wt. for *Ci*VCPO, IPTG 0.2 mM for *Lb*TDC, and nothing for *Ts*RTA), and the culture was shaken at 150 rpm, at the proper temperature for a defined amount of time (16 °C for 72 h for *Ci*VCPO, 25 °C for 4 h for *Lb*TDC, and 25 °C for 20 h for *Ts*RTA).

The cells were harvested by centrifugation (2500 RCF, 20 min, 4 °C), resuspended in loading buffer (see below) (2 mL/g of dry cell), and disrupted via sonication (10 min at 40% amplitude, 5 s ON + 5 s OFF cycles, using a FisherBrand sonicator). The cell debris was removed by centrifugation (12,100 RCF, 45 min, 4 °C), and the cell-free extract was filtered through a 0.45 μm pore-size syringe filter before purification via Ni-affinity chromatography for (*Lb*TDC and *Ts*RTA), while *Ci*VCPO required a different purification procedure: an equal amount of isopropanol was added to the cell-free extract to precipitate unstable proteins and nuclei acids that were removed by centrifugation (12,100 RCF, 30 min, 4 °C). The clear supernatant was loaded on a DEAE Sephadex A-25 column equilibrated with TRIS/H_2_SO_4_ (50 mM, pH 8.1). After washing the column with 2 volumes of TRIS/H_2_SO_4_ (50 mM, pH 8.1) and 2 volumes of 0.1 M NaCl in TRIS/H_2_SO_4_ (50 mM, pH 8.1), the enzyme was eluted with 1 M NaCl in TRIS/HCl (50 mM, pH 8.1). The fractions containing the desired protein were collected and subjected to dialysis (see Figures S9–S11 for SDS-PAGE of the protein expression and purification).

*Ci*VCPO: dialysis buffer TRIS/H_2_SO_4_ 50 mM + Na_3_VO_4_ 100 μM, pH 8.1.

*Lb*TDC: loading buffer: TRIS/HCl 25 mM + NaCl 300 mM + imidazole 20 mM + PLP 0.2 mM, pH 7.4; elution buffer: TRIS/HCl 25 mM + NaCl 300 mM + imidazole 280 mM + PLP 0.2 mM, pH 7.4; dialysis buffer: KPi 25 mM + NaCl 150 mM + PLP 0.2 mM, pH 7.4.

*Ts*RTA: loading buffer: KPi 50 mM + NaCl 100 mM + imidazole 30 mM + PLP 0.1 mM, pH 8.0; elution buffer: KPi 50 mM + NaCl 100 mM + imidazole 300 mM + PLP 0.1 mM, pH 8.0; dialysis buffer: KPi 50 mM + PLP 0.1 mM, pH 8.0.

### *Ci*VCPO Activity Assay

*Ci*VCPO (0.8 mg/mL, 20 μL) was added to the activity assay solution (monochlorodimedone 0.1 mM + H_2_O_2_ 10 mM + KBr 5 mM in citrate buffer 0.1 M, pH 5, 1 mL). The reaction was mixed at 30 °C for 3 min in a cuvette, and the depletion of monochlorodimedone was followed by registering the absorbance at 290 nm. One unit of *Ci*VCPO is defined as the amount of enzyme that catalyzes the depletion of 1 μmol/mL of monochlorodimedone in 1 min.

The immobilized *Ci*VCPO activity was measured in a similar way: the solid (50 mg) was suspended in the activity reagents’ solution (1 mL), and the reaction was stirred at room temperature for 10 min. At different reaction times (0, 1, 2, 3, 4, 6, 8, and 10 min), an aliquot of supernatant (40 μL) was taken and diluted 1:25 in citrate buffer (0.1 M, pH 5). The depletion of monochlorodimedone was followed by measuring the absorbance at 290 nm.

### *Lb*TDC Activity Assay

*Lb*TDC (0.1 mg/mL, 30 μL or 20 mg of the immobilized one) was added to the activity assay solution (l-tyrosine 2.5 mM + PLP 0.2 mM in AcONa 200 mM, pH 5, 1 mL). The reaction mixture was mixed at 37 °C for 20 min. At different reaction times (0, 5, 10, 15, and 20 min), an aliquot of reaction mixture (50 μL) was taken and diluted 1:8 in HCl (0.2% wt., 350 μL) to quench the reaction. The samples were subjected to HPLC analyses to monitor the l-tyrosine depletion.

One unit of *Lb*TDC is defined as the amount of enzyme that consumes 1 μmol/mL of l-tyrosine in 1 min.

### *Ts*RTA Activity Assay

*Ts*RTA (0.5 mg/mL, 20 μL) was added to the activity assay solution (sodium pyruvate 2.5 mM + (*R*)-methylbenzyl amine 2.5 mM + PLP 0.1 mM in KPi 50 mM + 0.25% DMSO, pH 8, 1 mL). The reaction mixture was stirred at 25 °C in a cuvette. Acetophenone formation was monitored by registering the absorbance at 245 nm for 4 min.

One unit of *Ts*RTA is defined as the amount of enzyme that produces 1 μmol/mL of acetophenone in 1 min.

The immobilized *Ts*RTA activity was similarly measured: the solid (20 or 50 mg) was transferred in a 15 mL Falcon tube and suspended in the activity reagents’ solution (5 mL for 20 mg or 10 mL for 50 mg of immobilized enzyme). The reaction mixture was stirred at room temperature with an orbital shaker for 10 min. Every minute, the supernatant (1 mL) was removed, its absorbance at 245 nm was measured, and it was readded to the reaction mixture.

### Immobilization on HAP-APTES with Glutaraldehyde

Hydroxyapatite derivatized with APTES (HAP-APTES, 20 mg) was suspended in a glutaraldehyde solution (GA, 10% wt., 2 mL) and mixed with an orbital shaker at room temperature for 1 h. During this period, the support turned from white to brown and finally to red. Then the mixture was filtered, and the solid was washed with the immobilization buffer (citrate 0.1 M, pH 5 for *Ci*VCPO, AcONa 200 mM + PLP 0.2 mM, pH 5 for *Lb*TDC, and KPi 50 mM + PLP 0.1 mM, pH 8 for *Ts*RTA, 3 × 1 mL). The solid was then resuspended in a solution of enzyme in immobilization buffer (different enzyme loading, 400 μL) and mixed with an orbital shaker at room temperature for 24 h (Figure S12).

The mixture was then filtered, and the solid was washed with immobilization buffer (2 × 400 μL). The protein concentration in both filtrate and washing was evaluated via Bradford assay, and the immobilized enzyme activity was measured.

### Immobilization on HAP-GLYMO Mediated by Co­(II) Ions

Hydroxyapatite derivatized with GLYMO (HAP-GLYMO, 50 mg) was suspended in modification buffer (iminodiacetic acid 2 M in sodium borate 0.1 M, pH 8, 100 μL) and stirred at room temperature for 2 h using an orbital shaker. Then the mixture was filtered, and the solid was washed with Milli-Q water (5 × 1 mL). The solid was resuspended in metal buffer (CoCl_2_ 30 mg/mL in Milli-Q water, 250 μL) and mixed at room temperature for 1 h during which it turned from white to purple. The mixture was then filtered, and the solid was washed again with Milli-Q water (5 × 1 mL) and then resuspended in an enzyme solution (different protein concentrations in KPi 50 mM, pH 8, 1 mL). The reaction was stirred at room temperature for 24 h (see Figure S13).

The mixture was then filtered again, and the solid was washed with desorption buffer (EDTA 50 mM + NaCl 0.5 M in KPi 50 mM, pH 7.2, 3 × 1 mL). The protein concentration in the filtrate and the washing was measured via Bradford assay.

The solid was resuspended in blocking buffer (l-glycine 3 M, pH 8.5, 200 μL) and stirred at room temperature for 20 h. The solid color became light pink. Finally, the mixture was filtered, and the immobilized enzyme was washed with Milli-Q water (3 × 1 mL) and activity assay buffer (AcONa 200 mM + PLP 0.2 mM, pH 5 for *Lb*TDC and KPi 50 mM + PLP 0.1 mM, pH 8 for *Ts*RTA, 3 × 1 mL). The immobilized enzyme activity was tested.

### Immobilization on HAP-GLYMO

HAP-GLYMO (20 mg) was suspended in an enzyme solution (different protein concentrations in KPi 50 mM, pH 8, 400 μL; see Figure S14). The reaction was mixed at room temperature for 24 h. Then the mixture was filtered, and the solid was washed with activity assay buffer (AcONa 200 mM + PLP 0.2 mM, pH 5 for *Lb*TDC and KPi 50 mM + PLP 0.1 mM, pH 8 for *Ts*RTA, 2 × 400 μL). The protein concentration in both filtrate and washing was measured via Bradford assay, and the activity of the immobilized enzyme was tested.

### Calculation of Immobilization Parameters

The percentage immobilization yield (IY) was calculated with the following equation

$$\mathrm{IY}=\frac{m_{i\mathrm{mmobilizedenzyme}}}{m_{\mathrm{offeredenzyme}}}\times 100=1-\frac{m_{\mathrm{enzymefiltrat}e}+m_{\mathrm{enzymewash}}}{m_{\mathrm{offeredenzym}e}}\times 100$$

where *m*_immobilizedenzyme_ (mg) is the mass of the enzyme immobilized, *m*_offeredenzyme_ (mg) is the mass of the enzyme given at the beginning of the immobilization process, *m*_enzymefiltrate_ and *m*_enzymewash_ (mg) are the mass of protein measured in the filtrate and washing, respectively.

The percentage recovered activity (RA) was calculated following the subsequent equation

$$\mathrm{RA}=\frac{\mathrm{Ac}t_{\mathrm{immobilizedenzyme}}}{\mathrm{Ac}t_{\mathrm{offeredenzyme}}\times \mathrm{loading}\times \mathrm{IY}}\times 100$$

where Act_immobilizedenzyme_ (U_immobilized_/g_support_) is the activity of the immobilized enzyme, Act_offeredenzyme_ (U_given_/mg_offeredenzyme_) is the specific activity of the enzyme offered at the beginning of the immobilization, loading (mg_enzyme_/g_support_) is the amount of given enzyme per gram of support, and IY is the immobilization yield.

### Reusability of Immobilized *Lb*TDC and *Ts*RTA

In order to measure the reusability of immobilized *Lb*TDC and *Ts*RTA, multiple consecutive activity assays were performed as described above. Between one reaction and the following one, the supernatant was removed either by filtration under reduced pressure or using a Pasteur pipette, and the immobilized enzyme was washed with the activity assay buffer (AcONa 200 mM + PLP 0.2 mM, pH 5, 2 × 1 mL for *Lb*TDC and KPi 50 mM + PLP 0.1 mM, pH 8, 3 × 3 mL for *Ts*RTA).

## Results and Discussion

### *Ci*VCPO Immobilization

Computational analyses of *Ci*VCPO superficial residues composition showed that 4.72% of the total surface area of the protein was made of lysines (i.e., 11 residues) that can be used for its immobilization (Figure 1).

**Figure 1:**
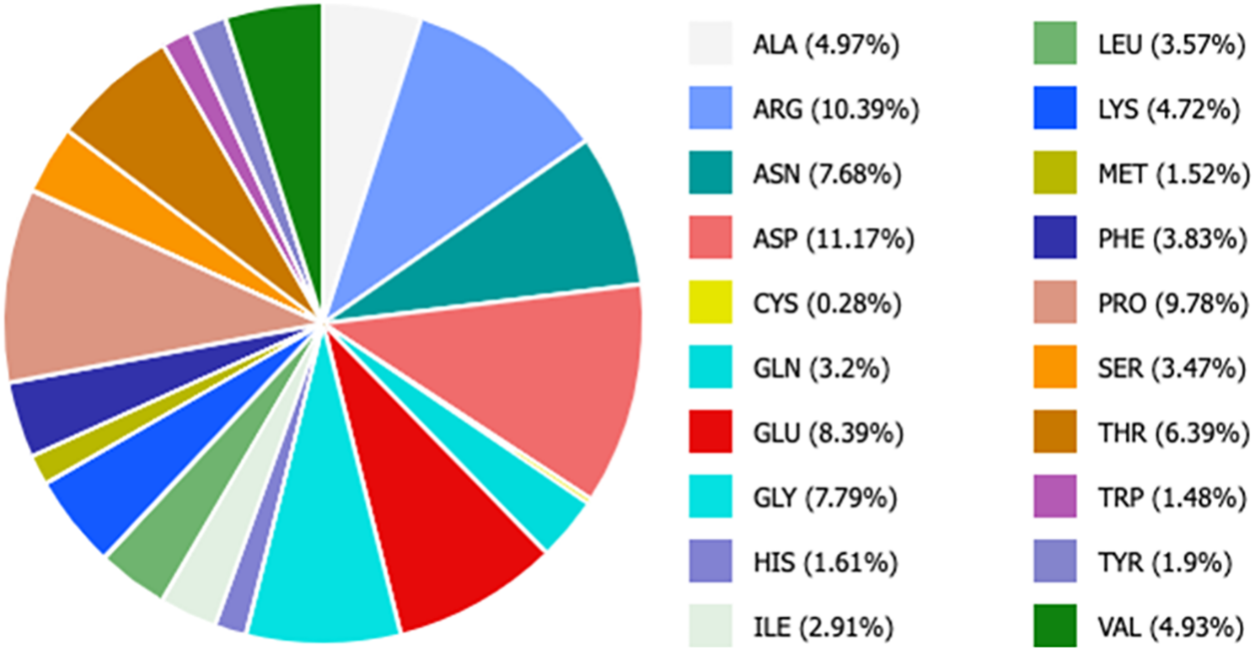
*Ci*VCPO surface analysis.

Different protein loadings were tested as shown in Table 1 but, despite reaching always high immobilization yield, comparable to the ones previously reported in literature with commercially available supports,^9^ the recovered activity of the immobilized enzyme always resulted in low values that decreased with higher protein loading.

**Table 1:** *Ci*VCPO Immobilization on HAP-APTES Using Glutaraldehyde as a Linker

enzyme loading (mg_enzyme_/g_support_)	IY (%)	RA (%)	immobilized activity (U/g_support_)
1	60	10	<0.2
2	74	8	<0.2
3	78	4	<0.2
5	75	2.1	<0.2

Although the decrease of recovered activity with the increase of protein loading is a common phenomenon in enzyme immobilization, in this case, this resulted in very poor immobilized activity, with values that never exceeded 0.2 U/g_support_. *Ci*VCPO was, therefore, not pursued further. Of course, we cannot exclude that a different immobilization strategy, e.g., the pretreatment of the enzyme with polyethylenimine and glutaraldehyde, could lead to better recovered activities; however, for consistency within our experimental approach, we did not explore further options.

### *Lb*TDC Immobilization on Derivatized HAP

*Lb*TDC, which also has been already immobilized on conventional support such as EP403/S, HFA403/S, and EP400/SS,^10^ was then investigated. In order to have results comparable to the ones reported in the literature, multiple immobilization approaches were trialed, namely, HAP-APTES-GA, used above with *Ci*VCPO, HAP-GLYMO + Co­(II) as reported in the literature, and HAP-GLYMO which exploits epoxy groups on the HAP for the direct immobilization with the lysine residues of the enzyme. (3-Glycidyloxypropyl)­trimethoxysilane (GLYMO) was used in the last two strategies to derivatize hydroxyapatite.

All strategies rely on lysine residues on the enzyme surface for immobilization chemistry. As shown in Figure 2, *Lb*TDC surface is very rich in lysine residues (69), accounting for 14.68% of the total area.

**Figure 2:**
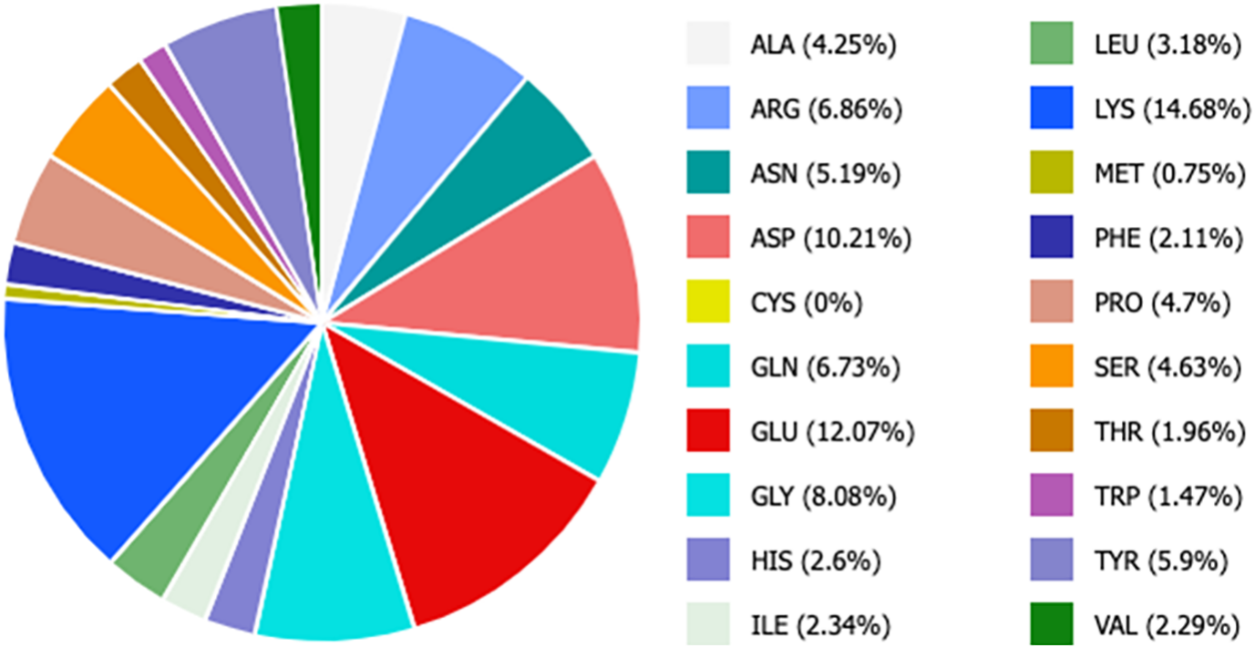
*Lb*TDC surface composition.

*Lb*TDC can be immobilized in high yields with all three strategies (Table 2) with peaks up to 94% when glutaraldehyde is employed. However, recovered activities reached a maximum of ∼17% and only at a low catalyst loading and low immobilization yield. With the GLYMO strategies, immobilization yields varied between ∼50 and ∼90%, but the recovered activity was less satisfactory, never exceeding 4%. While both HAP-APTES-GA and HAP-GLYMO gave at least measurable activities, they were lower than those reported in the literature with other supports.

**Table 2:** *Lb*TDC Immobilization with Different Techniques

	HAP-APTES-GA			HAP-GLYMO + Co(II)			HAP-GLYMO		
enzyme loading (mg_enzyme_/g_support_)	IY (%)	RA (%)	immobilized activity (U/g_support_)	IY (%)	RA (%)	immobilized activity (U/g_support_)	IY (%)	RA (%)	immobilized activity (U/g_support_)
1	20	17.1	2	72	1.6	0.5	60	9	3
2	80	5	4.2	79	1.1	0.8	80	4	3.2
3	90	3.5	5.1	67	1.1	1.0	73	3.9	4.1
5	94	2.2	4.4	51	1.1	0.9	82	2.8	4.8
10	82	1.2	4	58	0.8	1.6	89	1.7	6.5

### *Ts*RTA Immobilization

Finally, our focus shifted to *Ts*RTA. The stereoselective synthesis of amine remains a key target in organic chemistry, and the suitability of enzymes, in particular transaminases, has been amply demonstrated.^26^ *Ts*RTA is an excellent biocatalyst in this class, which can be produced with exceptionally high yields, up to 800 mg_enzyme_/L_culture_,^20^ and was therefore tested as a possible enzyme to be immobilized on HAP. The same three approaches used for *Lb*TDC were used with *Ts*RTA, which displays a lower content in superficial lysine residues, i.e., 30 lysines corresponding to 9.02% of the total surface of the dimer (Figure 3). The obtained results are summarized in Table 3.

**Figure 3:**
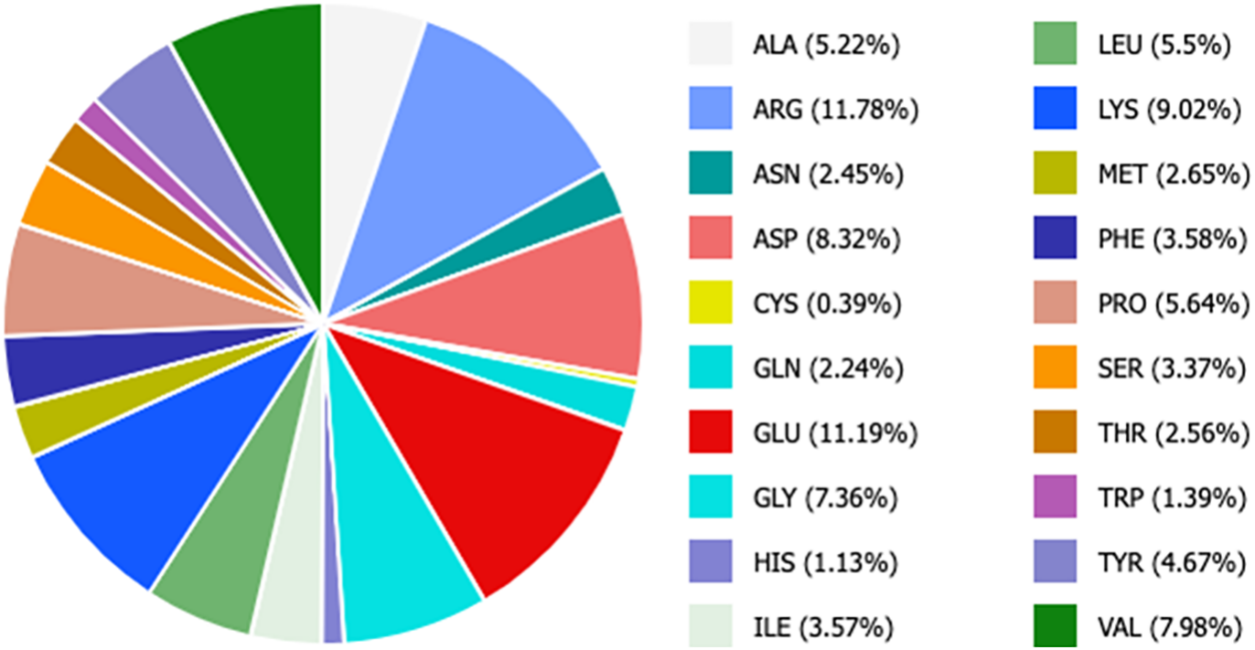
*Ts*RTA surface composition.

**Table 3:** *Ts*RTA Immobilization with Different Techniques

	HAP-APTES-GA			HAP-GLYMO + Co(II)			HAP-GLYMO		
enzyme loading (mg_enzyme_/g_support_)	IY (%)	RA (%)	Immobilized activity (U/g_support_)	IY (%)	RA (%)	immobilized activity (U/g_support_)	IY (%)	RA (%)	immobilized activity (U/g_support_)
1	80	50	0.6	0	n.d.	n.d.	0	n.d.	n.d.
2	95	40	1.4	20	n.d.	n.d.	0	n.d.	n.d.
3	94	47	2.4	0	n.d.	n.d.	10	n.d.	n.d.
5	94	38	3.5	20	n.d.	n.d.	0	n.d.	n.d.
10	98	26	4.8	8	n.d.	n.d.	0	n.d.	n.d.

As it can be noted, the strategy that makes use of glutaraldehyde allowed the achievement of high immobilization yields and good recovered activities that tend to decrease with protein loading higher than 3 mg_enzyme_/g_support_. On the other hand, it was not possible to obtain reproducible results using the GLYMO linker: only sometimes was it possible to achieve some immobilization exploiting the Co activation strategy, but the yields were always quite low, and therefore the recovered activities were not measured.

### Reusability of Immobilized *Lb*TDC and *Ts*RTA

One of the main goals pursued through enzyme immobilization is the possibility of reusing the biocatalyst in multiple reaction cycles. Therefore, following the screening of the different immobilization strategies, both *Lb*TDC and *Ts*RTA immobilized via APTES and glutaraldehyde, with a catalyst loading of 3 and 5 mg/g, respectively, were tested in multiple reaction cycles using their activity assay as model reactions. As reported in Figure 4, the immobilized activity of both enzymes showed a remarkable stability over six consecutive reactions, demonstrating that in all cases, once the enzyme is successfully immobilized, HAP is highly reliable with this strategy. A reduction step was not performed following cross-linking. In fact, the presence of the catalytic lysine, both *Lb*TDC and *Ts*RTA, which is bound to PLP in the resting state via a Schiff base, is incompatible with the reduction step. It is worth mentioning that despite this being a practice routinely applied (including in our group with suitable enzyme candidates), there is little evidence that such reduction improves the immobilized enzyme stability, and many different reaction mechanisms have been suggested over the years that do not imply the formation of a Schiff base.^27–29^

**Figure 4:**
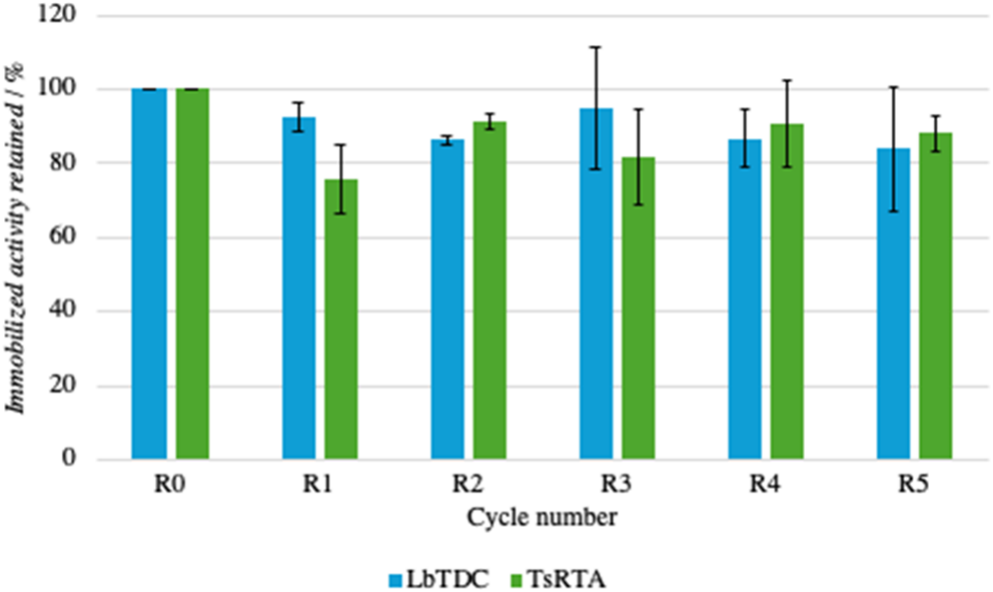
Reusability of *Lb*TDC and *Ts*RTA immobilized on HAP-APTES-GA over 6 consecutive reactions.

## Conclusions

In this work, the possibility of using hydroxyapatite as a support for covalent immobilization of three different enzymes was studied. Using APTES to functionalize the pristine support and glutaraldehyde as a linker, it was possible to immobilize all of the enzymes chosen with good immobilization yield. However, *Ci*VCPO presented a very low immobilized activity, while both *Lb*TDC and *Ts*RTA retained good activity upon immobilization, and it was possible to reuse them in six consecutive reactions with almost no deactivation.

On the other hand, the strategies that exploited GLYMO as a linker led to diverse results depending on the enzyme: in fact, *Lb*TDC was immobilized with comparable yield and recovered activity when a direct addition on the epoxide of the linker was performed, while both parameters gave lower values when the activation with cobalt­(II) ions was used. Instead, it was not possible to achieve decent immobilization of *Ts*RTA by exploiting GLYMO as a linker, neither through direct addition nor through cobalt­(II) activation.

## Supplementary Material



## Notes

^1^H NMR of the functionalized support, enzymes sequences and crystal stuctures, SDS-PAGE for all of the enzyme expression and purification, and schematic representation of the three immobilization strategies (PDF)
