# Supplementary material for: Covalent Immobilization of Different Enzymes on Hydroxyapatite, an Alternative Green Support
Source: ACS Omega. 2025 Sep 1;10(36):41899–905. doi: 10.1021/acsomega.5c06430 (PMC12444668; doi:10.1021/acsomega.5c06430)
Supplement: Supplementary file 1 [file ao5c06430_si_001.pdf]

Covalent immobilization of different enzymes on hydroxyapatite, an alternative green support

Leonardo Gelati[a,b,c] *, Antonella Gervasini*[a]*, Giovanna Speranza*[a]*, Francesca Paradisi*[c]*

[a] Department of Chemistry, University of Milan, via C. Golgi 19, 20139 Milan, Italy

[b] Department of Architecture and Industrial Design, University of Campania *Luigi Vanvitelli*, via San Lorenzo – Abazia di San Lorenzo, 81031 Aversa, Italy

[c] Department of Chemistry, Biochemistry and Pharmacology, University of Bern, Freiestrasse 3, 3012 Bern, Switzerland

Table of contents

NMR spectra 3
- **Figure S1.** ^{1}H-NMR spectrum of hydroxyapatite functionalized with APTES 3
- **Figure S2.** ^{1}H-NMR spectrum of hydroxyapatite functionalized with GLYMO 3

Enzyme sequences and crystal structures 4
- **Figure S3.** *Ci*VCPO sequence 4
- **Figure S4.** *Ci*VCPO crystal structure (pdb ID 1vni) with the lysine residues in evidence 4
- **Figure S5.** *Lb*TDC sequence 5
- **Figure S6.** *Lb*TDC crystal structure (pdb ID 5hsj) with lysine residues in evidence 5
- **Figure S7.** *Ts*RTA sequence 5
- **Figure S8.** *Ts*RTA crystal structure (pdb ID 6xwb) with lysine residues in evidence 6

Expression and purification of the three enzymes: *Ci*VCPO, *Lb*TDC, and *Ts*RTA 6
- **Figure S9.** SDS-PAGE for the purification of *Ci*VCPO 6
- **Figure S10.** SDS-PAGE for the purification of *Lb*TDC 7
- **Figure S11.** SDS-PAGE for the purification of *Ts*RTA 7

Strategies for covalent immobilization on hydroxyapatite 8
- **Figure S12.** Immobilization strategy with APTES and glutaraldehyde 8
- **Figure S13.** Immobilization strategy with GLYMO mediated by Co(II) ions 8
- **Figure S14.** Immobilization strategy with GLYMO 8

NMR spectra

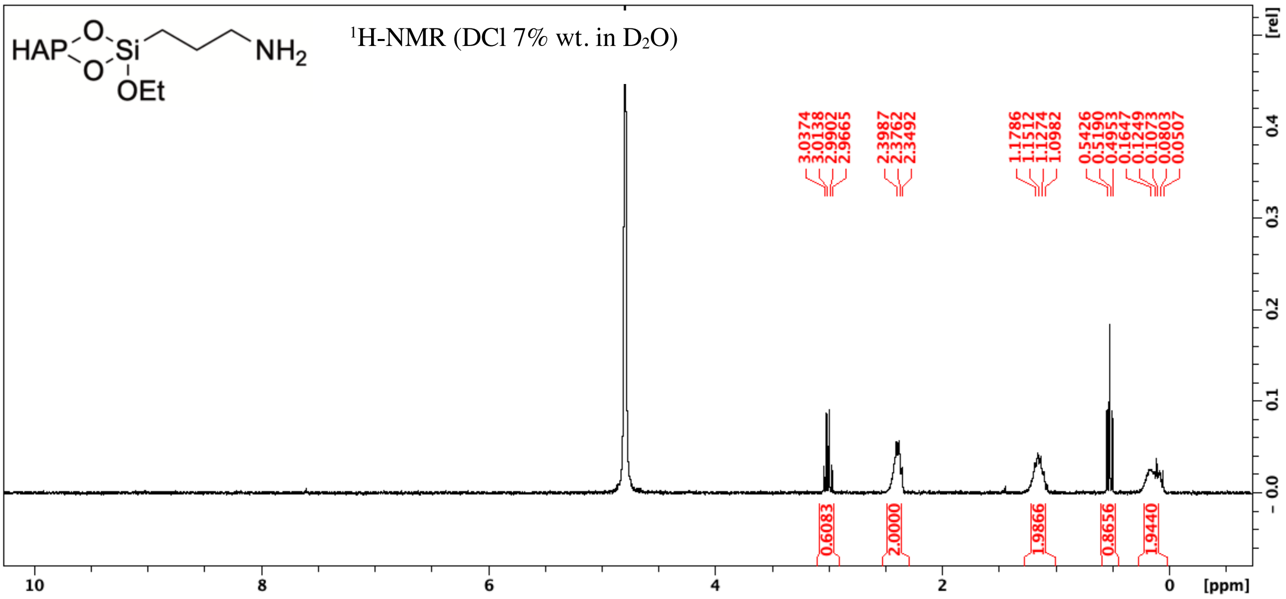

Figure S1. ^{1}H-NMR spectrum of hydroxyapatite functionalized with APTES

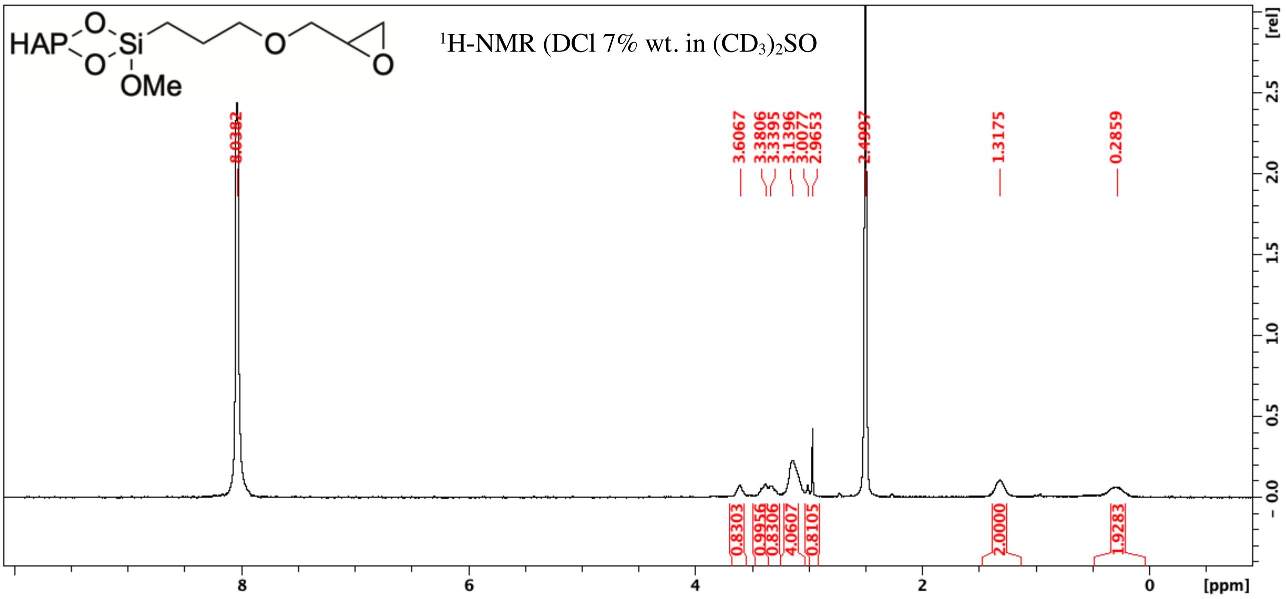

Figure S2. ^{1}H-NMR spectrum of hydroxyapatite functionalized with GLYMO

Enzyme sequences and crystal structures

```
          11         21         31         41         51          61         71
MGSVTPIPLP KIDEPEEYNT  NYILFWNHVG  LELNRVTHTV  GGPLTGPPLS  ARALGMLHLA  IHDAYFSICP  PTDF
        81         91         101        111        121         131         141
TTFLSP  DTENAAYRLP  SPNGANDARQ AVAGAALKML SSLYMKPVEQ PNPNPGANIS  DNAYAQLGLV LDRSVLEAP
  151        161        171        181        191         201        211        221
G GVDRESASFM  FGEDVADVFF ALLNDPRGAS QEGYHPTPGR YKFDDEPTHP VVLIPVDPNN PNGPKMPFRQ  Y
         231        241        251        261        271         281        291
HAPFYGKTT  KRFATQSEHF LADPPGLRSN ADETAEYDDA VRVAIAMGGA QALNSTKRSP WQTAQGLYWA YDGSNLI
    301        311        321        331        341        351        361
GTP  PRFYNQIVRR  IAVTYKKEED LANSEVNNAD FARLFALVDV  ACTDAGIFSW KEKWEFEFWR  PLSGVRDDGR
371        381        391        401        411        421        431        441
PDHGDPFWLT  LGAPATNTND  IPFKPPFPAY  PSGHATFGGA VFQMVRRYYN GRVGTWKDDE PDNIAIDMMI  SEELNG
    451        461        471        481        491         501        511
VNRD  LRQPYDPTAP  IEDQPGIVRT  RIVRHFDSAW  ELMFENAISR  IFLGVHWRFD  AAAARDILIP  TTTKDVYAVD
521        531        541        551        561        571
NNGATVFQNV  EDIRYTTRGT  REDEEGLFPI  GGVPLGIEIA  DEIFNNGLKP  TPPEIQPMPQ   ETPVQKPVGQ   Q
PVKGMWEEE   QAPVVKEAP
```

Figure S3. *Ci*VCPO sequence

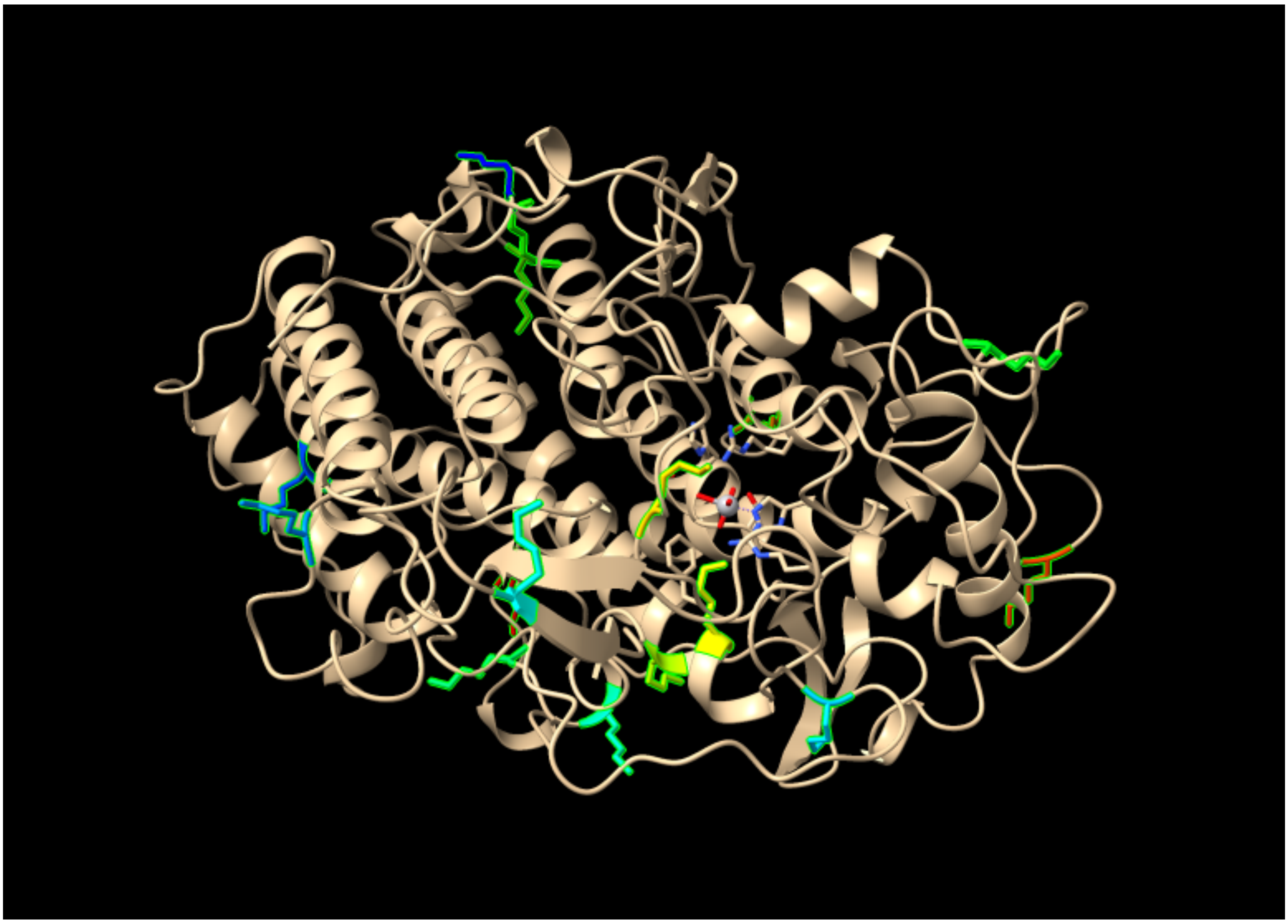

Figure S4. *Ci*VCPO crystal structure (pdb ID 1vni) with the lysine residues in evidence

```
               11               21              31              41              51               61
MEKSNRSLKD   LDLNALFIGD   KAENGQLYKD   LLNKLVDEHL   GWRKNYIPSD   PNMIGPEDQN   SPAFKKTVGH
71              81               91              101             111             121             131             141
MKTVLDQLSE   RIRTESVPWH   SAGRYWGHMN   SETLMPALLA   YNYAMLWNGN   NVAYESSPAT   SQMEEEVGQE   FARL
          151             161             171             181             191             201             211
MGYDYG   WGHIVADGSL   ANLEGLWYAR   NIKSLPFAMK   EVNPELVAGK   SDWELLNMPT   KEIMDLLENA   GSQIDEVKKR
221             231             241             251             261             271             281             291
SARSGKNLQR   LGKWLVPQTK   HYSWMKAADI   IGIGLDQVVP   VPIDSNYRMD   IQALESIIRK   YAAEKTPILG   VVGVAG
       301             311             321             331             341             351             361
STEE   GAVDGIDKIV   ALRQKLQKEG   IYFYLHVDAA   YGGYARALFL   DEDDQFIPYK   NLQKVHAENH   VFTEDKEYIK
371             381             391             401             411             421             431             441
PEVYAAYKAF   DQAESITIDP   HKMGYVPYSA   GGIVIQDIRM   RDTISYFATY     VFEKGADIPA   LLGAYILEGS   KAG
         451             461             471             481             491             501             511
ATAASVW   AAHHTLPLNV   TGYGKLEGAS   IEGAHRYYDF   LKNLKFEVAG   KRISVHPLIS   PDFNMVDYVL   KEDGNDDLI
    521             531             541             551             561             571             581             591
E   MNRLNHAFYE   QASYVKGSLY   GKEYIVSHTD   FAIPDYGDSP   LAFVESLGFS   EVEWRHAGKV   TIIRASVMTP   YMN
           601             611
QRENFDY   FAPRIKKAIQ   ADLEKVYASV     NQKENVLEHH     HHHH
```

Figure S5. *Lb*TDC sequence

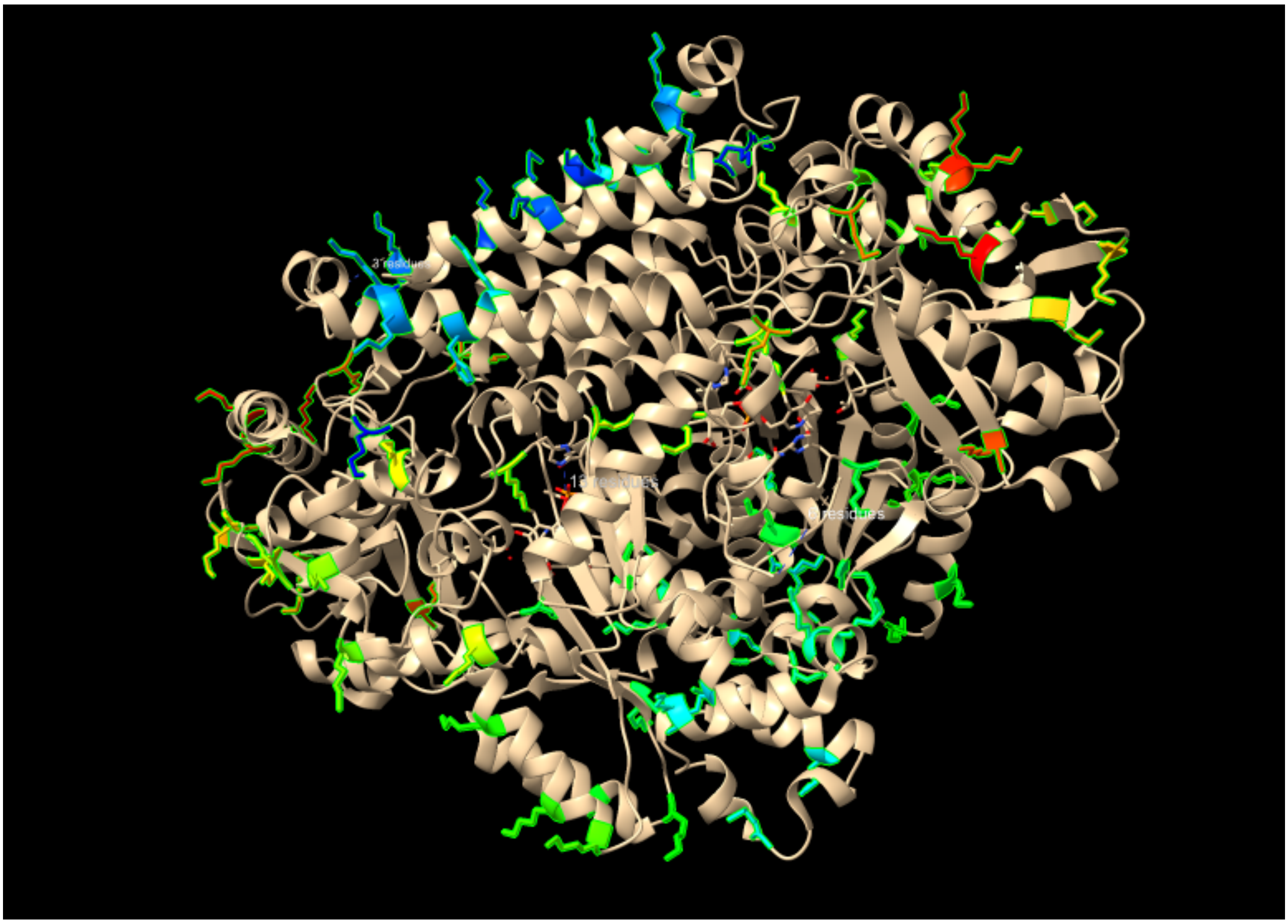

Figure S6. *Lb*TDC crystal structure (pdb ID 5hsj) with lysine residues in evidence

```
                2               12              22              32              42              52
MDIGINSDPM   ATMDKVFAGY   AARQKAMEAA   GNPLSEGIAW   VEGEMVPLHE   ARIPMLDEGF   MRSDLTYDVP
62              72              82              92              102             112             122             132
SVWDGRFFRL   DDHLSRLEAS   CAKLRLKLPL   PREEVKKILV   EMVAKSGIRD   AFVEIIVTRG   LKGVRGSRPE   EIV
         142             152             162             172             182             192             202
NRLYMLV   QPYVWVMEPE   VQPVGGDAVI   ARTVRRVPPG   SIDPTVKNLQ   WGDFVRGLFE   ASDRGATYPF   LTDGDANLT
   212             222             232             242             252             262             272             282
E   GSGFNIVLVK   DGVLYTPDRG   VLQGVTRKSV   IDVANAKGFE   VRVEYVPVEA   AYHADEIFMC   TTAGGIMPIR   SLD
         292             302             312
GKPVNDG   KVGPITKAIW   DGYWEMHYDP   AYSFEIKYQV     AEGKPLAGYR     FQEKLAAAEN     LYFQGLEHHH     H

HH
```

Figure S7. *Ts*RTA sequence

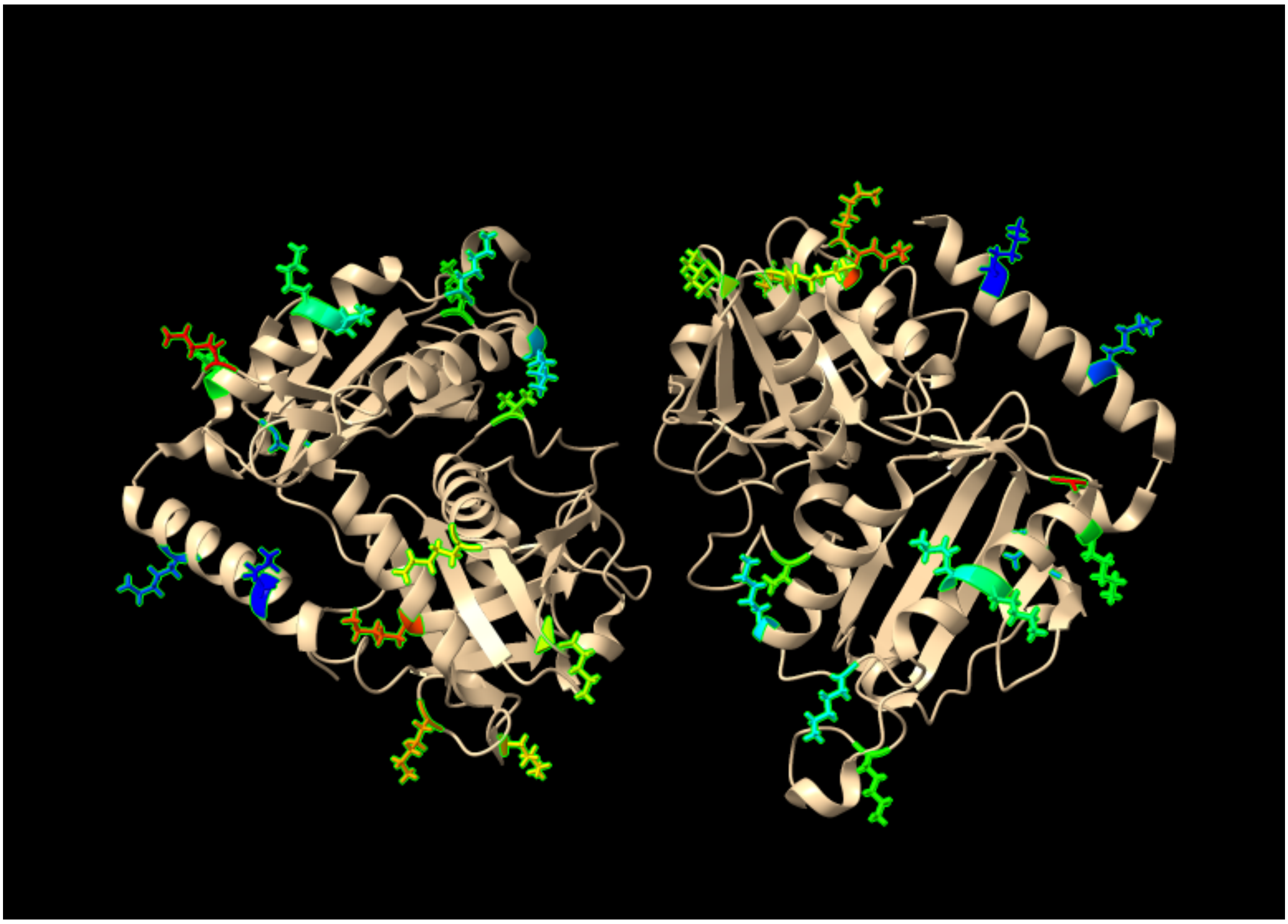

Figure S8. *Ts*RTA crystal structure (pdb ID 6xwb) with lysine residues in evidence

Expression and purification of the three enzymes: *Ci*VCPO, *Lb*TDC, and *Ts*RTA

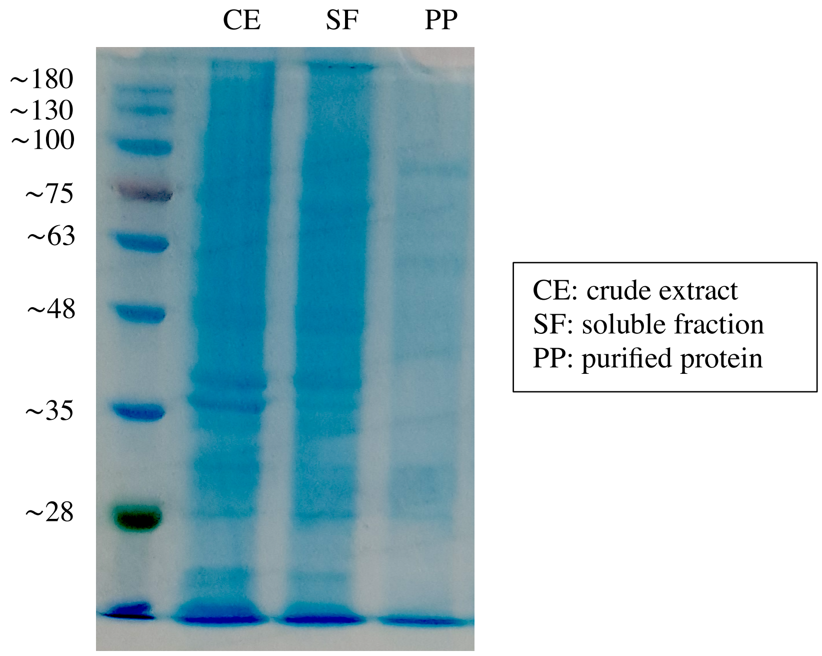

Figure S9. SDS-PAGE for the purification of *Ci*VCPO

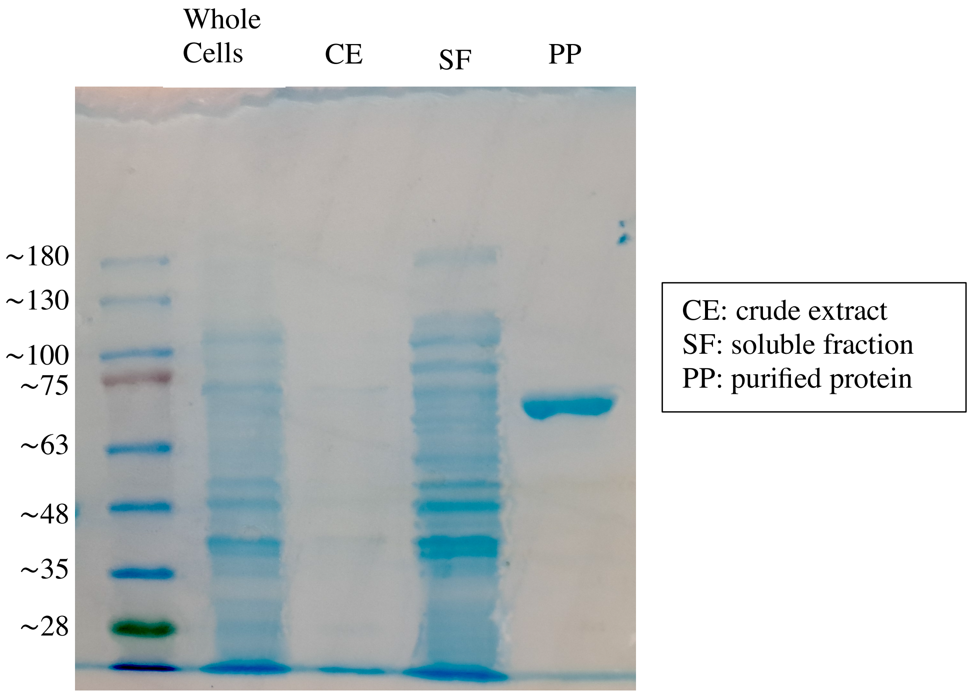

Figure S10. SDS-PAGE for the purification of *Lb*TDC

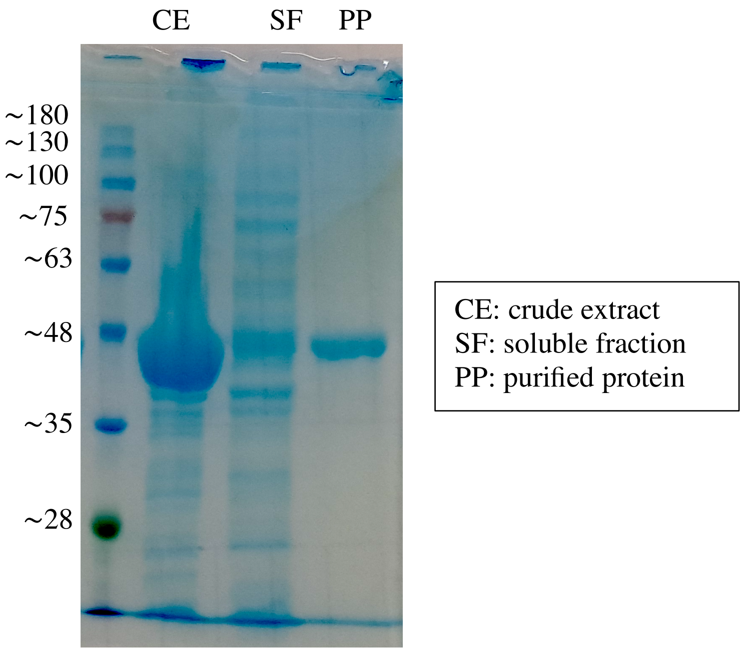

Figure S11. SDS-PAGE for the purification of *Ts*RTA

Strategies for covalent immobilization on hydroxyapatite

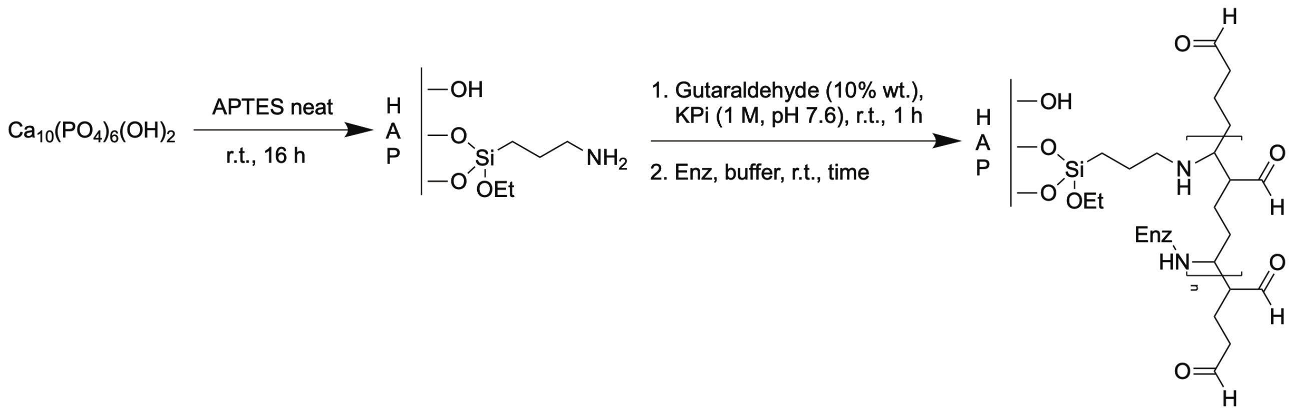

Figure S12. Immobilization strategy with APTES and glutaraldehyde

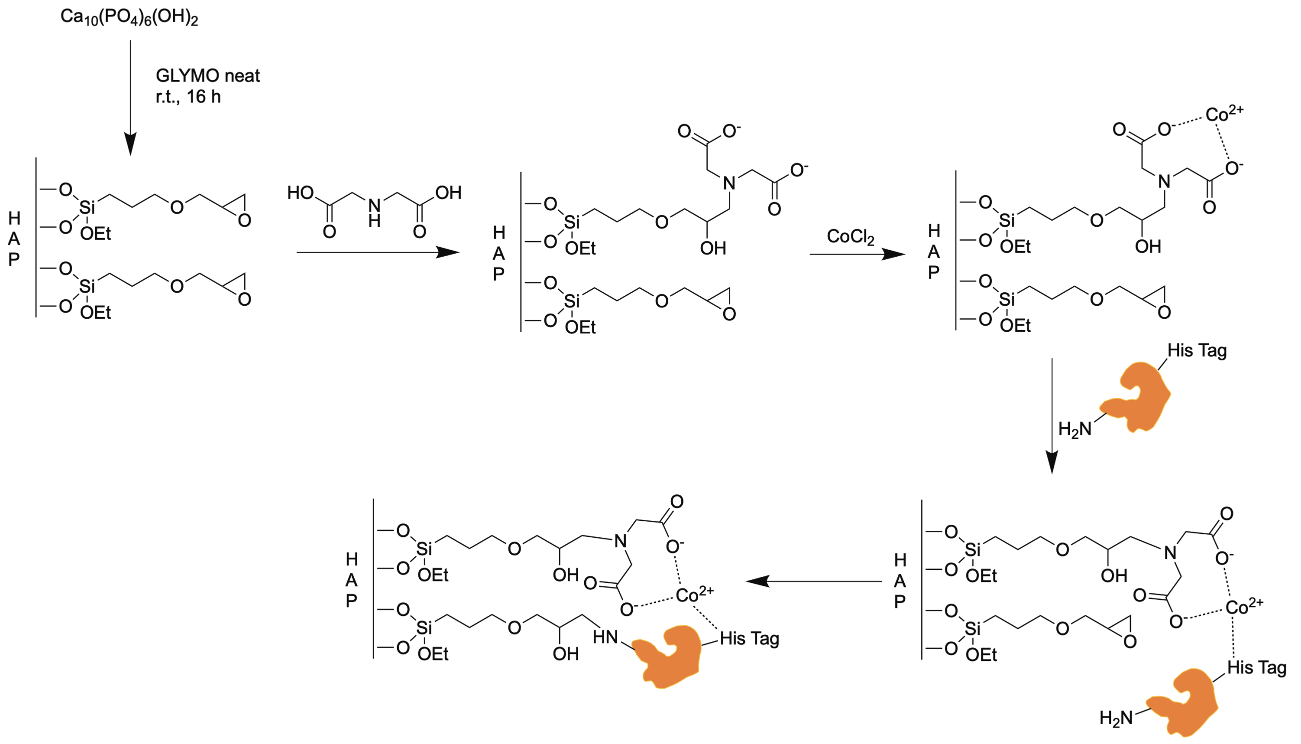

Figure S13. Immobilization strategy with GLYMO mediated by Co(II) ions

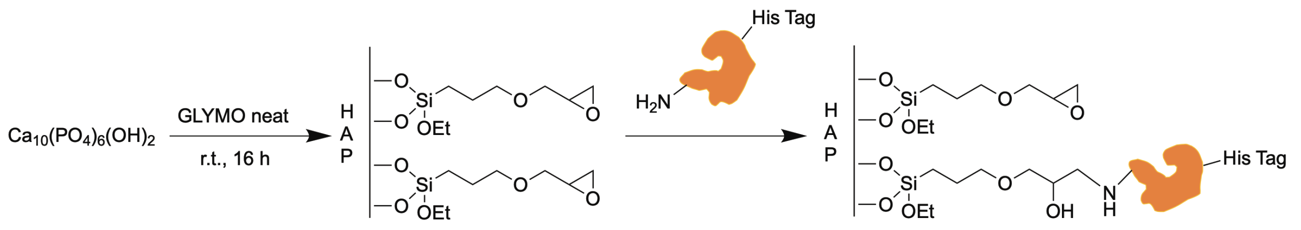

Figure S14. Immobilization strategy with GLYMO
